# Personalised Dosimetry in Nuclear Medicine: Bridging Physics, Biology and AI for Next Generation Radiopharmaceutical Therapy

**DOI:** 10.1007/s13139-026-00988-8

**Published:** 2026-03-11

**Authors:** Joycie Shanmugiah, Jin Su Kim

**Affiliations:** 1 Division of Applied RI, Korea Institute of Radiological and Medical Sciences (KIRAMS), 75 Nowon-ro, Nowon-gu, Seoul, 01812 Republic of Korea; 2 Radiological and Medical Sciences, University of Science and Technology (UST), Seoul, Republic of Korea

**Keywords:** PRRT, ^177^Lu, Dosimetry, Nuclear medicine, SPECT/CT, Artificial intelligence, Organ-level MIRD, Voxel based dosimetry

## Abstract

Radiopharmaceutical dosimetry is rapidly evolving from a physics-dominated calculation tool to a central pillar of precision nuclear medicine. As targeted radionuclide therapies expand across indications, there is a growing clinical imperative to personalize dose estimation, predict therapeutic efficacy and mitigate organ toxicity. This review critically examines the current landscape of dosimetry methods including organ level Medical Internal Radiation Dose (MIRD) schema, voxel-based S-values, Monte Carlo (MC) simulations and emerging artificial intelligence (AI)-assisted segmentation tools and their translational relevance. Through a comprehensive literature search of ^177^Lu peptide receptor radio nuclide therapy (PRRT) studies published between 2020 and 2025, we evaluate methodological heterogeneity and quantify dose variations across organs. Findings reveal persistent inconsistencies in absorbed dose estimates with reported kidney doses varying, 0.3–0.9 Gy/GBq and tumor doses ranging 1–10 Gy/GBq largely driven by differences in imaging protocol timing, segmentation strategy, and time-point sampling across studies. We also discuss regulatory trends, biologically informed dosimetry models incorporating relative biological effectiveness (RBE), and future integration with dose-point kernel (DPK) based and dose-volume histogram (DVH) driven computational frameworks. The field must now shift toward harmonized, reproducible standards that bridge physics, biology, and computation, transforming dosimetry into a predictive engine for individualized radiopharmaceutical therapy (RPT).

## Introduction

### The Paradigm Shifts in Nuclear Medicine Dosimetry

Nuclear medicine has undergone a paradigm shift from a primarily diagnostic field to a cornerstone of precision oncology and personalized therapy. At the heart of this transformation lies dosimetry, which quantitatively measures the radiation absorbed dose at organ, tissue, and cellular levels. Traditionally, therapeutic radiopharmaceuticals were administered based on fixed or empirically derived activity prescriptions, often without accounting for patient-specific pharmacokinetics or biodistribution [1]. Recent clinical evidence has demonstrated a definitive dose-response relationship in radionuclide therapies. In ^177^Lu-DOTATATE peptide receptor radionuclide therapy (PRRT), tumor-absorbed doses above approximately 100 Gy are consistently associated with improved progression-free and overall survival, whereas kidney toxicity remains uncommon below cumulative exposures of 23 Gy or 0.5–0.9 Gy/GBq per cycle when amino-acid protection is used. Bone-marrow suppression is rarely observed unless total absorbed doses exceed about 2 Gy. In selective internal radiation therapy (SIRT) with ^90^Y microspheres, mean absorbed doses of 70–75 Gy to normal liver for glass and ≤ 40 Gy for resin microspheres are considered safe thresholds, while higher tumor doses (> 100 Gy) correlate with better local control. For prostate specific membrane antigen (PSMA)-targeted therapies, salivary-gland doses typically range 0.4–1.0 Gy/GBq, with xerostomia risk increasing at the upper end of this range. These quantitative benchmarks are summarized in Table 1, highlighting representative dose–response and toxicity thresholds that underscore the clinical necessity for individualized, dosimetry-guided treatment planning to optimize efficacy while safeguarding organs at risk. These findings have driven a transition toward individualized, dosimetry-guided treatment planning, with the goal of maximizing tumor control while reducing toxicity to healthy tissues [2].

**Table 1:** Representative absorbed-dose thresholds for tumor response and organ toxicity in radiopharmaceutical therapy Gy/GBq: Gray per gigabecquerel; NET: neuroendocrine tumor; OS: overall survival; PFS: Progression-free survival; PRRT: peptide receptor radionuclide therapy; PSMA: Prostate-specific membrane antigen; TARE: Trans-arterial radioembolization

Organ/Target	Threshold Range	Endpoint	Reference
Tumor (NET, PRRT)	100 Gy (response threshold); higher response > 175 Gy	Improved PFS / OS	[3]
Kidney	23 Gy (total); 0.5–0.9 Gy/GBq per cycle	Nephrotoxicity	[4–7]
Bone marrow	2 Gy (total)	Hematologic toxicity	[8]
Liver (^90^Y TARE non-tumoral)	Glass:70–75 Gy; Resin ≤ 40 Gy (non-tumoral liver)	Hepatotoxicity	[9, 10]
Salivary glands (PSMA)	0.4–1.0 Gy/GBq (range up to 2.5 Gy/GBq)	Xerostomia	[11, 12]

Gy/GBq: Gray per gigabecquerel; NET: Neuroendocrine tumor; OS: Overall survival; PFS: Progression-free survival; PRRT: Peptide receptor radionuclide therapy; PSMA: Prostate-specific membrane antigen; TARE: Trans-arterial radioembolization

### Beyond Physics: Towards a Multidisciplinary Framework

Traditionally, dosimetry has been based on physics-driven models, such as the Medical Internal Radiation Dose (MIRD) schema, organ-level S-values, and voxel-based dose calculations derived from quantitative Single Photon Emission Computed Tomography/Computed Tomography (SPECT/CT) and Positron Emission Tomography/Computed Tomography (PET/CT) imaging [13, 14]. While these methodologies have provided a robust framework, they are often constrained by assumptions of homogeneous radiopharmaceutical distribution and limited biological specificity. Emerging therapies, particularly those involving alpha-emitting radionuclides and nanoparticle-based agents, require more sophisticated approaches that incorporate microdosimetry and considerations of relative biological effectiveness (RBE) [15]. Moreover, advances in artificial intelligence (AI) offer promising avenues to automate image quantification, enhance dosimetric accuracy, and develop predictive dose-response models. For example, DeepDoseNet, a deep learning-based tool, has demonstrated high accuracy in predicting voxel-level absorbed dose distributions from SPECT images [16–18]. Collectively, the integration of physics, biology, and AI represents the foundation of next-generation radiopharmaceutical therapy (RPT).

### Clinical Imperatives for Advancing Dosimetry

The clinical impetus for enhanced dosimetry arises from multiple factors: the need to accurately predict therapeutic efficacy and minimize toxicity risks [19]; the demand for individualized treatment regimens aligned with the theranostic paradigm [20]; regulatory frameworks emphasizing dosimetry-based therapy planning, such as the European Association of Nuclear Medicine (EANM) guidelines (2015) and the International Commission on Radiation Units and Measurements (ICRU) Report 96 [21]; and the expanding indications for RPT, including alpha-emitters, bispecific targeting agents, and combination therapies [22]. Despite these drivers, several challenges persist. Standardization across centers remains limited [23]. Quantification uncertainties, including PVE and image registration errors, continue to compromise dosimetric accuracy [24]. Additionally, workflow complexity and resource demands impede widespread clinical adoption [25]. Addressing these challenges requires a coordinated, interdisciplinary approach involving nuclear medicine physicians, medical physicists, radiobiologists, and computational scientists [26]. This review aims to provide a comprehensive and integrative perspective on personalized dosimetry in nuclear medicine, emphasizing the convergence of physics, biology, and AI. By examining both established and emerging dosimetric methodologies, we highlight the necessity of incorporating biological endpoints into dose-response modeling and explore how AI-driven approaches can revolutionize image quantification and predictive modeling. Furthermore, we discuss current clinical applications, identify persistent challenges, and propose a pragmatic roadmap to translate these multidisciplinary advances into routine practice. Ultimately, this review envisions dosimetry evolving from a physics-centric calculation framework into a biologically informed, AI-enhanced clinical decision-making tool, thereby solidifying its role as a cornerstone of precision nuclear medicine.

Despite the clear clinical rationale for individualized dosimetry, its widespread implementation remains hindered by several technical and logistical barriers. Quantification accuracy is limited by partial-volume effects, which lead to underestimation of activity concentration in small lesions or organs with dimensions less than two to three times the system’s full width at half maximum (FWHM) [24, 27].Additional uncertainties arise from image-registration errors between functional and anatomical datasets caused by respiratory motion, bowel peristalsis, or inconsistent patient positioning during serial imaging [28].Limited time-point sampling also remains a major constraint: most clinical protocols acquire only two or three post-therapy scans, which restricts accurate time–activity curve fitting and increases error in cumulated-activity estimation [29, 30].Furthermore, inter-operator variability in segmentation and the absence of standardized VOI delineation criteria contribute to significant dose variations across institutions [31, 32]. Finally, resource and staffing limitations continue to impede clinical adoption, as manual segmentation and multi-time-point data processing require extensive physicist time and infrastructure support [33, 34]. Collectively, these challenges underscore the need for harmonized acquisition protocols, automation of quantification and segmentation workflows, and dedicated training programs to enable reproducible and scalable dosimetry in everyday nuclear-medicine practice.

## Physics Backbone: Dosimetry Methodologies in Nuclear Medicine

### MIRD Schema and Organ- Level Dosimetry

The MIRD formalism has historically served as the backbone of internal dosimetry, relying on pre-calculated S-values derived from mathematical phantoms and assuming uniform activity distribution within defined source and target organs [1, 13, 35]. Tools such as Organ Level INternal Dose Assessment/EXponential Modeling (OLINDA/EXM) have streamlined its implementation and continue to be employed in regulatory settings, particularly for iodine therapy, bone-seeking radiopharmaceuticals, and early PRRT applications [36–38]. Despite its operational simplicity, the MIRD model is inherently constrained by key assumptions. It treats organ uptake as homogeneously distributed, a simplification that does not reflect the heterogeneity observed in actual patient anatomy and biodistribution patterns. Furthermore, the use of reference phantoms precludes patient-specific accuracy, and the model fails to accommodate the short-range emissions critical in alpha-particle therapy [24, 29, 33]. More recent extensions of the MIRD schema attempt to improve anatomical realism by integrating hybrid SPECT/CT workflows; however, the methodology remains fundamentally limited in resolution and personalization [39].

The MIRD approach, based on simplified S-value modeling, has remained a foundational reference in internal dosimetry but shows a relatively stable publication pattern over 2005–2025. In contrast, studies referencing organ-level dosimetry, whether explicitly using that term or describing it through related phrases, have demonstrated a marked increase in frequency, particularly after 2015. This temporal shift underscores the field’s growing emphasis on improving anatomical resolution in absorbed dose calculations and moving toward patient-specific planning in therapeutic nuclear medicine. The trend aligns with advancements in hybrid imaging, segmentation software, and regulatory pressures for individualized dosimetric assessment in therapies such as PRRT, SIRT, and PSMA-targeted treatments.

While the conventional MIRD schema relies on reference-phantom S-values derived from standardized anatomical models, such as those implemented in OLINDA/EXM, patient-specific dosimetry employs individualized S-values calculated from actual CT-based organ geometries and voxel density maps. The difference between these two approaches can be substantial: studies have shown organ-to-organ absorbed-dose discrepancies of 15–40% when substituting reference-based coefficients with patient-specific values, primarily due to variations in organ size, mass, and inter-organ distance [1].

Despite these limitations, the MIRD approach remains appropriate for several practical applications, particularly in screening or epidemiological studies where population-level estimates suffice and patient-specific data are unavailable, in pediatric dosimetry when CT acquisition is challenging or infeasible, and in circumstances where computational resources or imaging data for individualized modeling are constrained [13, 24, 40, 41].

### Voxel-Based Dosimetry: the Shift Toward Patient Specificity

The emergence of voxel-based dosimetry marked a significant advancement in internal radiation dose assessment. By leveraging quantitative three-dimensional imaging from SPECT/CT or PET/CT, along with anatomical density maps from CT, this approach enables absorbed dose calculation on a voxel-wise basis [27, 42]. Time-integrated activity maps are generated from serial imaging, which are then converted into dose distributions using voxel S-values or dose-point kernels (DPKs). The resulting spatial resolution facilitates precise dose estimation and allows generation of dose-volume histograms (DVHs), drawing conceptual parallels with external beam radiotherapy planning.

Voxel-based dosimetry has proven particularly valuable in clinical scenarios such as PRRT with ¹⁷⁷Lu-DOTATATE, where lesion-specific dosimetry has demonstrated predictive power for tumor response and therapeutic outcome [43–45]. In liver-directed therapies like SIRT using ⁹⁰Y microspheres, voxel-level assessments account for intrahepatic heterogeneity and provide more accurate organ-at-risk evaluation compared to organ-averaged models [46–48]. More recently, its application has extended to PSMA-targeted radioligand therapy, combining PET-based quantification with high-resolution segmentation to capture uptake variations across salivary glands, kidneys, and metastases [49, 50]. Clinical integration of voxel-based methods is supported by commercially available software platforms including Hybrid Dosimetry Module™ from HERMES, STRATOS™ from Philips, and PLANET^®^ OncoDose from DOSIsoft, which offer user-friendly interfaces and semi-automated workflows [51]. The EANM has published procedural guidelines to support standardization and encourage multi-center adoption [52].

### Monte Carlo Simulation (MC): Accuracy at a Computational Price

MC simulations represent the gold standard in dosimetric precision by modeling the stochastic transport of individual particles through heterogeneous anatomical geometries. These simulations comprehensively account for physical interactions such as scattering, absorption, and energy deposition across both soft and hard tissue matrices [53, 54]. Patient-specific voxelized geometries, derived from CT datasets, can be incorporated into MC engines such as GATE [55], MCNP [56], EGSnrc [57], and Geant4 [58, 59], enabling detailed simulation of complex biodistributions and tissue-specific energy deposition. MC-based dosimetry has been pivotal in elucidating the microdosimetric properties of alpha-emitting therapies, such as ^225^Ac-PSMA and ^213^Bi-DOTATOC, where the short path length of emissions necessitates sub-organ resolution for accurate modeling [60]. Moreover, MC methods serve as a benchmark for validating voxel-based dosimetric approximations and are indispensable in preclinical and research contexts where maximal dosimetric accuracy is required. Despite their precision, the clinical implementation of MC techniques remains constrained by practical limitations. Simulations are computationally intensive, often requiring access to high-performance computing clusters or GPU acceleration. Additionally, substantial expertise in radiation physics and computational modeling is needed, posing a barrier to routine clinical adoption. Ongoing efforts aim to mitigate these challenges through computational optimization and the development of AI-based surrogate models trained on MC outputs, offering potential for real-time clinical application [61].

Although MC transport remains the reference standard for internal dosimetry accuracy, it is computationally intensive. Typical patient-specific SPECT/CT-based simulations using GATE, Geant4, or EGSnrc involve 10^7^-10^9^ primary particle histories to achieve acceptable statistical uncertainty (< 3–5%) for voxel dose maps [53, 55]. On a modern workstation (Intel i9 or comparable CPU) such runs require 2–8 h for a single-organ or partial-body geometry; full-body patient models can extend to 12–24 h depending on image resolution and variance target. GPU-accelerated and distributed implementations have reduced these times by roughly one order of magnitude, enabling near-real-time generation of low-noise dose maps for clinical feasibility studies.

To mitigate runtime and statistical noise, several variance-reduction techniques (VRTs) are routinely applied. These include Russian roulette and splitting to optimize particle weighting, forced-collision and photon-forcing to enhance scoring in low-probability regions, and importance sampling based on energy or spatial biasing [59, 60]. For voxel-level simulations, adaptive stratification and track-length estimators further reduce variance without altering physical realism. Collectively, these strategies can decrease computation time by 50–80% while preserving dose accuracy within ± 2% relative to fully analog simulations.

Such methodological advances, coupled with hybrid AI-surrogate models trained on MC data, are progressively transforming MC dosimetry from a purely research tool into a clinically deployable framework capable of patient-specific dose prediction within practical timeframes.

### Image Quantification and Practical Limitations

The reliability of any dosimetric methodology is intrinsically linked to the quantitative imaging. However, various technical and biological factors introduce quantification errors that compromise accuracy. One of the most prominent technical limitations is the partial volume effect (PVE), which stems from the finite spatial resolution of SPECT and PET systems. This phenomenon systematically underestimates radioactivity concentrations in small structures, especially when lesion diameters are less than two to three times the system’s FWHM [62]. Misregistration between functional and anatomical modalities such as PET and CT can further degrade spatial fidelity. This issue is particularly pronounced in organs subject to motion, including the liver and lungs, where respiratory motion, bowel peristalsis, and variations in patient positioning frequently led to alignment errors [28, 63]. Another limitation lies in the sampling density of time–activity curves. Optimal dosimetry requires multiple timepoints to accurately estimate cumulated activity through integration. However, clinical constraints often restrict image acquisition to only two or three timepoints, thereby limiting the robustness of pharmacokinetic modeling and absorbed dose estimation [29]. Image reconstruction algorithms play a pivotal role in PET-based dosimetry, as they directly influence recovery coefficients (RCs), image uniformity, and absorbed dose calculations [64, 65]. Recent advances in image reconstruction methodologies offer promising solutions. Techniques such as Bayesian inference and penalized likelihood reconstruction have demonstrated improved signal-to-noise ratios while preserving spatial resolution [66]. In parallel, AI-based segmentation tools, particularly those leveraging convolutional neural networks (CNNs), are being employed to enhance reproducibility in volume-of-interest (VOI) delineation and minimize inter-operator variability (Weisman, Kieler et al. 2020).

To mitigate inter-institutional variability and improve reproducibility, standardization initiatives have been introduced by expert groups. Recommendations from the EANM Dosimetry Committee [67–69] and ICRU Report 96 [21] emphasize harmonized acquisition protocols, calibration using standardized phantoms, and unified reporting frameworks to reduce systematic uncertainties across different imaging platforms and clinical centers.

In internal dosimetry, total absorbed-dose uncertainty results from cumulative errors in calibration, image quantification, time–activity integration, and dose-conversion processes, typically amounting to 20–40% for MIRD-based and 15–25% for optimized voxel-based methods. Additional uncertainty in DVHs arises from voxel resampling and image registration, highlighting the importance of adhering to EANM Dosimetry Committee recommendations for standardized uncertainty reporting [24, 27, 30].

Quantitative image recovery strongly depends on the relationship between object size and the system’s spatial resolution. The RC, defined as the ratio between measured and true activity concentration, decreases steeply for structures smaller than approximately two to three times the FWHM of the imaging system. For modern SPECT/CT systems used in ^177^Lu therapy, the spatial resolution at 208 keV typically ranges 7–10 mm FWHM, yielding RCs of 0.9 for 30 mm spheres, 0.6 for 15 mm, and 0.3–0.4 for 10 mm objects when reconstructed with standard OSEM protocols and without partial-volume correction [24, 27]. PET-based dosimetry with ^68^Ga or ^90^Y shows similar trends, with partial-volume loss exceeding 50% for lesions smaller than twice the system FWHM [62].

Motion artifacts further degrade quantification accuracy and consequently the spatial fidelity of dose maps. Respiratory motion in thoraco-abdominal imaging can cause blurring of several millimetres-comparable to the system’s intrinsic resolution-leading to underestimation of lesion activity by 10–20% and distortion of organ boundaries. Bowel peristalsis introduces additional misregistration between sequential SPECT and CT acquisitions, particularly affecting liver and intestinal dosimetry. Prospective respiratory gating, phase-matched CT acquisition, and data-driven motion correction algorithms have been shown to substantially reduce these artifacts and improve quantification consistency [28, 63]. Implementing partial-volume correction factors together with motion-management techniques therefore remains essential for improving quantitative accuracy and reproducibility of internal-dosimetry studies.

### Evolution Toward Personalized Physics Workflows

The field of internal dosimetry is steadily advancing toward truly personalized workflows that integrate hybrid imaging, model-based inference, and automation. Recent studies have demonstrated the clinical feasibility of patient stratification based on individualized organ and lesion-specific absorbed doses, enabling informed treatment escalation or de-escalation strategies. Dose-toxicity modeling for organs at risk - particularly the kidneys, bone marrow, and salivary glands—is now being incorporated into clinical protocols, moving beyond conventional fixed-activity prescriptions and enabling therapy optimization [70]. AI-enhanced tools are accelerating this transformation. Deep learning architectures such as U-Net and DeepDoseNet have shown promise in automating organ segmentation, predicting three-dimensional dose distributions, and synthesizing dose maps from limited input datasets [32, 71, 72]. These models offer MC-level accuracy with significantly reduced computational demands, facilitating the translation of high-precision dosimetry into routine clinical practice.

**Fig. 1:**
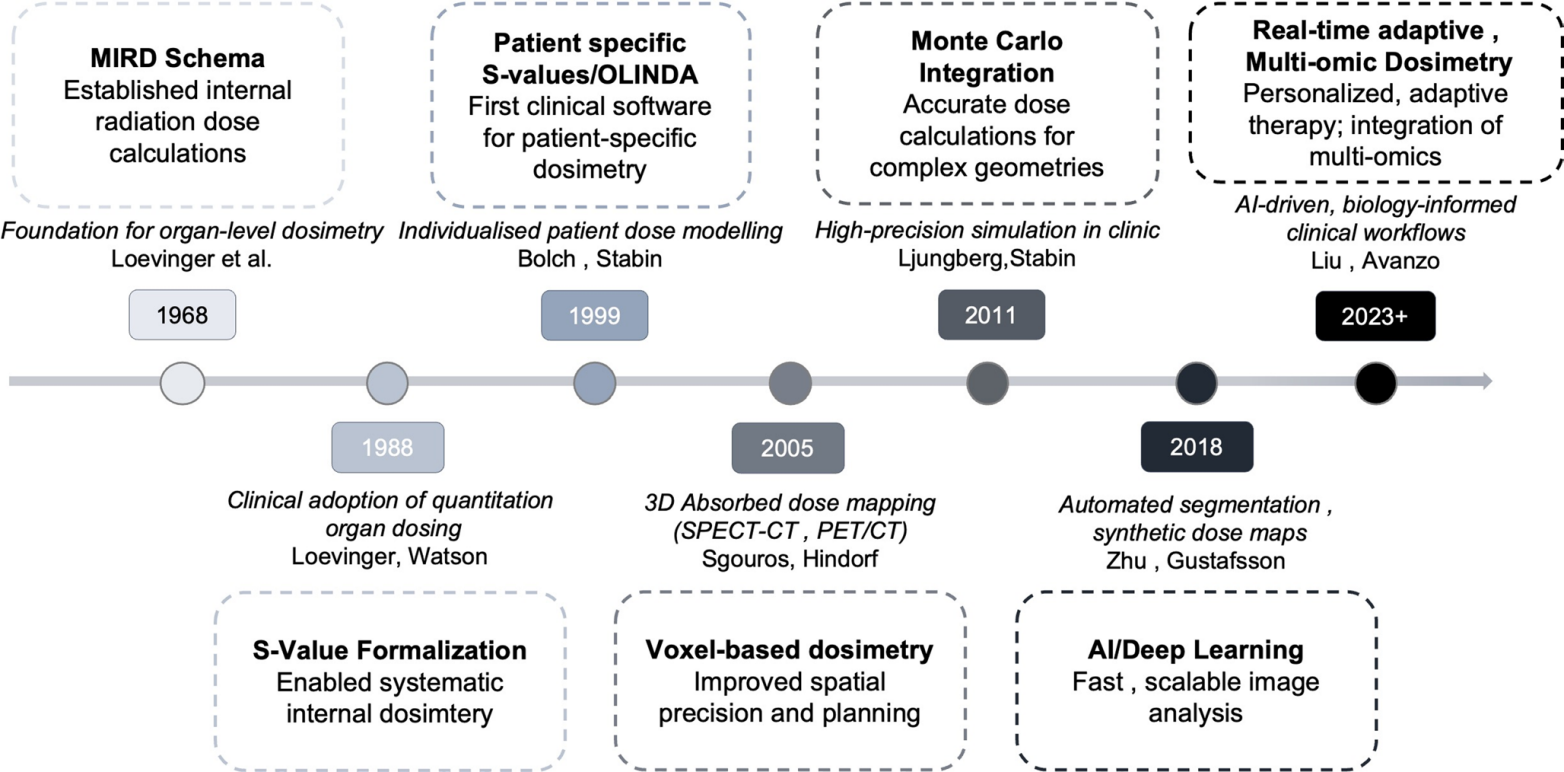
**Evolution of personalized dosimetry in Nuclear Medicine** This timeline summarizes major technological and conceptual advances that have shaped the field of internal dosimetry

The integration of patient-specific imaging, voxel-level modeling, and AI-assisted inference marks a paradigm shift from physics-limited frameworks to real-time, adaptive dosimetric planning. This evolution positions nuclear medicine within the broader context of precision oncology. Figure 1 summarizes the major milestones in internal dosimetry, from the introduction of the MIRD schema (1968) and S-value formalism (1988), through the development of patient-specific tools such as OLINDA (1999) and the transition to voxel-based dosimetry (2005). The incorporation of MC simulations (2011) enabled high-resolution dose estimation in anatomically complex settings, while the advent of AI-based methods (2018) further enabled automated segmentation and synthetic dose generation. Most recently, the convergence of multi-omics data with real-time adaptive dosimetry (2023-present) signals the onset of a fully personalized, biology- and AI-driven era in radiopharmaceutical therapy.

### Dosimetry Workflow, Uncertainty Propagation, and DVH Considerations

Quantitative voxel-based dosimetry typically follows a four-step workflow comprising (i) scanner calibration, (ii) activity recovery, (iii) time-integrated activity estimation, and (iv) absorbed-dose conversion. During scanner calibration, system-specific sensitivity factors derived from phantom measurements are applied to convert reconstructed image counts into absolute activity values (Bq). Activity recovery involves corrections for attenuation, scatter, and partial-volume effects to restore the true activity distribution within organs or lesions. The time-integrated activity is obtained by fitting decay-corrected time–activity curves from serial quantitative SPECT or PET acquisitions to calculate the cumulated activity per voxel. Finally, absorbed-dose conversion is achieved using DPK convolution, voxel S-value scaling, or MC transport methods to generate patient-specific 3D dose maps.

The accuracy of internal dosimetry depends on how uncertainties propagate through each stage of the calculation workflow-from scanner calibration to final dose conversion. Quantitative imaging introduces the first layer of variability, where calibration factors, cross-calibration with dose calibrators, and system normalization collectively contribute approximately 2–5% uncertainty [24]. The next step, activity recovery, is affected by partial-volume effects, scatter correction, and attenuation modeling, with uncertainties ranging from 5 to 10% in large organs to as high as 30% for sub-centimeter lesions [27]. Integration of time-activity data represents another dominant error source; limited time sampling or bi-exponential fitting inaccuracies can introduce 15–25% variability in cumulated activity, particularly in single-time-point (STP) approaches [30]. Finally, conversion of cumulated activity to absorbed dose through DPK or voxel S-value scaling contributes an additional 5–10%, depending on whether reference or patient-specific geometry is applied. When propagated cumulatively, these factors result in a total organ-level uncertainty of approximately 20–40% for MIRD-based methods and 15–25% for optimized voxel-based or MC workflows.

Beyond these analytical uncertainties, the interpretation of DVHs is subject to specific pitfalls. DVHs derived from voxel-based dose maps are sensitive to resampling bias, particularly during image co-registration or interpolation between SPECT and CT grids, which can distort the high-dose tail and underestimate small-volume hotspots [24]. Furthermore, smoothing and discretization during dose-map reconstruction may artificially reduce apparent heterogeneity within tumors. Therefore, DVHs should be interpreted with consideration of voxel resolution, motion artifacts, and segmentation uncertainty, and wherever possible, validated against MC or high-fidelity phantom simulations.

Recognizing and reporting these uncertainty components has become a key requirement under the EANM Dosimetry Committee and ICRU Report 96 recommendations, ensuring harmonized uncertainty quantification and improved comparability of absorbed-dose data across institutions.

## Biological Dimension: Integrating Biology into Dosimetry

### Rationale for Biological Integration

Traditional dosimetry in nuclear medicine, rooted in the MIRD schema and voxel-based methods, has prioritized the physical quantification of absorbed dose. However, clinical evidence shows that equivalent absorbed doses may elicit heterogeneous biological responses depending on tissue characteristics, tumor microenvironment, radiation quality, and individual radiosensitivity [73–77]. These discrepancies underscore the necessity of incorporating biological endpoints into dosimetric assessments to enable truly personalized radiopharmaceutical therapy. Bridging physical and biological dosimetry not only refines therapeutic efficacy but also enhances safety by improving toxicity prediction [78].

### RBE in Nuclear Medicine Therapy

RBE quantifies the biological potency of different radiation types relative to a standard, typically ^60^Co gamma rays. Alpha-emitting radionuclides such as ^223^Ra and ^225^Ac, which exhibit high linear energy transfer (LET), demonstrate RBE values often 3–7 times higher than beta emitters, reflecting their enhanced capacity for inducing complex DNA damage [79–81]. Crucially, RBE is not an intrinsic constant-it varies with cell type, dose rate, oxygenation status, and genetic background, complicating its clinical application. Ongoing efforts aim to define therapy- and patient-specific RBE values, with regulatory and clinical frameworks evolving to accommodate RBE-adjusted dosimetry.

### DNA Damage Biomarkers: γ-H2AX and DNA Double-Strand Breaks (DSB) Repair

Absorbed dose is an imperfect surrogate for biological damage, as it does not directly quantify DNA injury, the principal driver of radiotoxicity and therapeutic effect. The phosphorylated histone variant γ-H2AX serves as a sensitive biomarker for DSBs, enabling the quantification of radiation-induced genomic damage [82–84]. Quantifying γ-H2AX foci and analyzing DSB repair kinetics provide insight into cellular radiosensitivity and establish a link between physical dosimetry and biological outcomes. In PRRT, for example, sustained γ-H2AX elevation in peripheral blood lymphocytes correlates with hematologic toxicity and reduced therapeutic efficacy [85].

### Radiogenomics: Molecular Fingerprinting and Predictive Modeling

Radiogenomics seeks to associate dosimetric parameters with individual genomic and transcriptomic profiles that modulate radiosensitivity, DNA repair capacity, and toxicity risk [86, 87]. Integrating genomic alterations such as mutations in DNA repair genes into dosimetric models enables improved risk stratification and individualized dosing. Multi-omic and AI-driven platforms are now being developed to fuse physical, biological, and molecular datasets, opening new avenues for predictive modeling in precision radiotherapy [88, 89].

### Microdosimetry and cellular-level Dose Deposition

Microdosimetry characterizes stochastic energy deposition at the scale of individual cells or subcellular compartments, a crucial consideration for radiopharmaceuticals emitting short-range particles such as alpha particles or Auger electrons [90]. Advanced techniques such as track-structure MC simulations and in vitro microdosimetric assays are being employed to predict cellular responses with a resolution beyond organ- or voxel-based dosimetry [91], Despite their potential, translational challenges remain, including limited spatial resolution of in vivo imaging and the need for individualized biological input.

Biology-integrated dosimetry represents the next frontier in radionuclide therapy, combining spatially resolved physical dose data with mechanistic biological modeling. In a seminal study, Tamborino et al. applied GATE Monte Carlo-based microdosimetry in NCI-H69 xenograft mice, correlating voxel-level absorbed dose with γ-H2AX-marked DSBs and in vivo survival [92]. Their findings revealed a strong linear relationship between local dose and DSB induction (0.022 DSB/cell/mGy, R² = 0.7) and demonstrated that heterogeneous receptor uptake can result in up to 59% higher biological dose compared to uniform models. Radiobiological modeling incorporating DNA repair dynamics and tumor repopulation achieved optimal fit parameters (α = 0.1 Gy⁻¹, α/β = 100 Gy), highlighting the distinct radiobiology of PRRT compared to external beam radiotherapy. These insights affirm the critical role of biology-integrated dosimetry in optimizing patient-specific outcomes in molecular radiotherapy. The reported 0.022 DSB cell⁻¹ mGy⁻¹ originates from NCI-H69 (SSTR2⁺) calibration and is not universally applicable. DSB yield for ^177^Lu varies with β-spectrum, dose-rate, cell geometry, subcellular localization, and DNA-repair kinetics, often differing by 2-3-fold across models. To link voxel-based dosimetry with biological effect, patient-specific S-values (MIRD_cell_, IDAC-Cell, Geant4-DNA) are applied to downscale doses to the cellular level, separating self- and cross-dose by cellularity and spacing. Intratumoral heterogeneity is addressed through stochastic or cluster-based activity models, and biologic response is derived via LQ modeling with dose-rate (G-factor) correction. Non-uniformity is summarized using micro-DVHs or gEUD (generalized Equivalent Uniform Dose), with sensitivity analysis on internalization and repair parameters to bound uncertainty.

## AI and Machine Learning in Dosimetry

### Introduction: the Digital Revolution in Dosimetry

The advent of AI and machine learning (ML) has ushered in a transformative era in nuclear medicine dosimetry. These technologies are reshaping the field from labor-intensive, manual procedures to automated, scalable, and data-driven approaches [93]. Beyond enhancing workflow efficiency and standardization, AI enables the identification of complex, non-linear relationships among imaging data, absorbed dose metrics, biological parameters, and clinical outcomes. Consequently, AI is emerging as a key enabler of personalized and adaptive radiopharmaceutical therapy.

### Deep Learning for Organ Segmentation and Quantification

Accurate segmentation of organs and lesions is fundamental to dosimetry, as it directly affects absorbed dose estimations. Manual segmentation is time-consuming, prone to inter- and intra-observer variability, and requires considerable expertise. In recent years, CNNs, notably U-Net architectures, have demonstrated superior performance in automated segmentation across SPECT, PET, and CT imaging modalities [17, 94]. Trained on expertly annotated datasets, these models consistently outperform traditional atlas-based and region-growing algorithms, offering rapid, reproducible, and high-fidelity delineations [95]. The adoption of such AI-driven segmentation tools has significantly advanced the implementation of robust and individualized dosimetry workflows.

### AI-driven Dose Estimation and Synthetic Dose Mapping

**Fig. 2:**
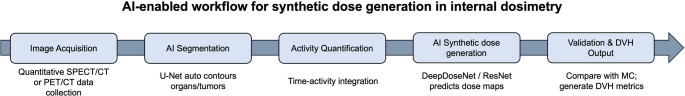
**Schematic overview of the AI-assisted dosimetry pipeline. **Quantitative imaging data (SPECT/CT or PET/CT) are processed through AI-based segmentation models to generate organ and lesion masks. These segmented volumes are used for activity quantification and input to deep-learning models (e.g., DeepDoseNet) that predict synthetic absorbed-dose distributions. Predicted dose maps are then compared with voxel-based or Monte Carlo reference calculations to produce DVHs and biological outcome metrics

Beyond segmentation, AI techniques are increasingly applied to the direct estimation of three-dimensional absorbed dose distributions. Recent deep learning models can generate synthetic dose maps [96] using limited input data-such as single time-point SPECT images or incomplete time-activity curves [97]. For instance, architectures like DeepDoseNet have shown promising accuracy in absorbed dose prediction comparable to MC simulations, but with drastically reduced computational time [98]. These innovations hold promise for real-time adaptive dosimetry and allow for dynamic dose recalculations in response to intra-therapy changes or patient-specific variations. The integration of AI into dosimetry workflows enables rapid and standardized quantification of absorbed doses by automating key image-processing steps. Figure 2 illustrates a typical workflow in which AI contributes to both organ segmentation and synthetic dose prediction. The process begins with acquisition of quantitative SPECT/CT or PET/CT images following therapeutic administration. These datasets undergo AI-based segmentation, typically using CNNs such as U-Net or transformer-based architectures trained on manually annotated ground-truth volumes. Automated segmentation replaces labor-intensive manual contouring, ensuring reproducible region-of-interest (ROI) delineation for organs and tumors. The resulting segmented volumes feed directly into activity quantification and time–activity curve generation.

For synthetic dose estimation, deep-learning models such as DeepDoseNet or VoxelDose-ResNet learn nonlinear mappings between limited input data (e.g., a single post-therapy SPECT scan or a pre-therapy PET scan) and reference MC dose distributions. These models are trained using paired datasets consisting of input images and corresponding MC-derived dose maps. Once trained, the AI model can predict voxel-wise absorbed-dose maps in seconds, achieving MC-like accuracy (MAE = 0.05–0.1 Gy/GBq; RMSE = 0.1–0.2 Gy/GBq) with significantly reduced computation time. The resulting synthetic dose maps are then validated against conventional voxel or MC calculations and exported to generate DVHs or biological response parameters. This integrated AI-assisted workflow substantially reduces operator dependence, improves reproducibility, and facilitates near real-time personalized dosimetry.

### Predictive Analytics: Dose-Response Modeling and Toxicity Risk

Machine learning methods, including random forests, support vector machines, and deep ensemble models, are increasingly utilized to model dose-response relationships and to predict treatment efficacy and toxicity risk [99]. By integrating multimodal datasets including imaging, dosimetry metrics, clinical data, and radiogenomic information-AI models can reveal complex interactions that may elude conventional statistical approaches. Applications in PRRT [100] and SIRT [101] have demonstrated the superior predictive capabilities of AI-based approaches over purely physics-driven models.

Recent AI-based dosimetry studies have leveraged quantitative imaging datasets linking absorbed dose to biological and clinical outcomes. Typical cohorts include 80–250 patients for model training, with 20–30% for validation and 15–20% for independent testing, derived from quantitative ¹⁷⁷Lu-DOTATATE or ¹⁷⁷Lu-PSMA-617 SPECT/CT and ⁶⁸Ga- or ⁹⁰Y-PET/CT acquisitions reconstructed using OSEM or PSF recovery [96, 102].

Predictive models such as gradient boosting, residual CNNs, and transformer networks use voxel- or region-based dosimetric features (mean dose, D70, gEUD, micro-DVH metrics) to forecast tumor response and organ-specific toxicity. Dose maps from Monte Carlo or voxel-based reference models serve as training labels, with standardized preprocessing including decay correction, voxel resampling (2–4 mm), and z-score normalization to ensure consistency across patients and scanners.

To mitigate domain shifts from differences in hardware, reconstruction, or protocol, studies employ (i) data augmentation to mimic scanner noise and resolution changes, (ii) SUV-based normalization to harmonize intensity scales, (iii) transfer learning and domain-adversarial training to generalize across vendors, and (iv) cross-institutional validation. These methods consistently achieve MAE = 0.1–0.2 Gy/GBq and AUC > 0.85 for toxicity prediction, supporting the robustness of AI-based dose–response analytics across imaging platforms [16, 18]. Such predictive frameworks represent a critical step toward biologically informed precision dosimetry, integrating absorbed-dose distributions with clinical and molecular data to estimate patient-specific efficacy and toxicity risk.

Several deep-learning models have now achieved MC-like accuracy in voxel-level absorbed-dose estimation. Reported mean absolute errors (MAE) range from 0.05 to 0.15 Gy/GBq, and root mean square errors (RMSE) from 0.08 to 0.20 Gy/GBq, relative to MC ground truth across organs and tumors [16, 18, 42, 96]. When assessed by DVH metrics, voxelwise AI-predicted doses show < 5% deviation in D50 and D70 values and maintain gEUD differences below 3%, confirming clinical equivalence.

In terms of computational efficiency, full MC simulations typically require 4–12 h on a standard CPU workstation (10⁸-10⁹ particle histories), whereas AI-based surrogate models produce synthetic dose maps within 1–5 s on a single GPU (NVIDIA RTX 3090 or equivalent), or under 1 min on standard CPU hardware. These accelerations up to 10³-10⁴ fold faster enable near–real-time dose prediction without significant loss of accuracy. Such performance validates the use of AI-driven predictive dosimetry as a reliable and computationally efficient complement to conventional MC frameworks, particularly for prospective dose-response modelling and adaptive radionuclide therapy.

### Automation and Integration of Dosimetry Workflows

The most transformative potential of AI lies in the automation and full integration of the dosimetry pipeline. Emerging end-to-end platforms now connect image acquisition, organ segmentation, quantitative analysis, dose estimation, toxicity prediction, and clinical reporting within a cohesive framework. Such automation facilitates standardized dosimetry even in resource-constrained environments and supports the clinical realization of adaptive, patient-specific radiopharmaceutical therapies.

### Challenges: Data Scarcity, Validation, and Interpretability

Despite these advances, significant barriers remain. Chief among them is the limited availability of large, standardized, and multicentric datasets, which hinders the development and validation of generalizable models. Additionally, the “black box” nature of most deep learning algorithms raises concerns regarding interpretability and clinical trust. Current research is increasingly focused on developing explainable AI (XAI) models, establishing robust validation protocols, and facilitating data-sharing initiatives across institutions.

### The Future: Explainable, Adaptive, and Multi-Modal AI Dosimetry

Future directions point toward the development of transparent, adaptive AI systems that integrate dosimetry, biological, and multi-omics data for real-time clinical decision support. Standardized frameworks for data curation, model validation, and regulatory oversight will be critical to the safe and effective clinical deployment of AI-enabled dosimetry in nuclear medicine.

## Time Point-Based Dosimetry: STP and MTP Approaches in Clinical PRRT and RLT

Accurate quantification of organ and tumor absorbed doses is essential for personalized radiopharmaceutical therapy. The current gold standard is multi-time point (MTP) dosimetry, which entails acquiring serial quantitative SPECT/CT or planar images at multiple time points after radiopharmaceutical administration. This approach enables detailed characterization of pharmacokinetics and accurate calculation of the area under the time-activity curve (AUC), leading to patient-specific dose estimations [103]. However, the clinical adoption of MTP dosimetry is often limited by logistical and patient-related challenges. It is resource-intensive, requires repeated imaging, and may be impractical for patients with limited mobility or requiring multiple treatment cycles.

In response, single time point (STP) dosimetry has emerged as a pragmatic alternative. In STP protocols, a single post-therapy scan is acquired typically at 24- or 72-hours post-injection and mathematical models or population-based kinetic parameters are used to extrapolate the full time–activity curve [104]. Common methods involve applying population-derived correction factors [105] or bi-exponential decay models [106]. Although STP reduces patient and institutional burden, its accuracy can be compromised in individuals with atypical kinetics, renal impairment, or altered clearance. Consequently, while STP provides clinically useful approximations, MTP remains the preferred approach when precise dose estimation is critical such as in dose-escalation trials or for patients at high risk of toxicity. Ongoing advancements in AI-driven pharmacokinetic modeling and the expansion of population-based dosimetry databases are expected to enhance the accuracy and applicability of STP dosimetry. These innovations may ultimately support the broader implementation of personalized dosimetry in routine clinical practice.

## Clinical Translation and Impact

### Quantitative Impact and Workflow Efficiency of Personalized Dosimetry

Voxel-based or AI-assisted dosimetry pipelines have markedly improved efficiency and standardization compared with traditional semi-manual MIRD workflows. Automated pipelines reduce total processing time from 4 to 6 h to 30–45 min per patient, a 70–90% reduction and achieve Dice similarity coefficients of 0.88–0.95 for automated segmentation reproducibility [96, 102, 107]. In prospective PRRT studies, dose-adjusted treatment cycles reduced cumulative renal absorbed dose by 20% and limited grade ≥ 3 hematologic toxicity to < 5% without compromising tumor control [44]. Retrospective analyses in PSMA-targeted therapy reported 20–30% inter-cycle dose variability, showing that adaptive planning can preserve therapeutic tumor dose while minimizing salivary and marrow exposure [108].The integration of AI-assisted workflows across centers further improved harmonization, achieving intra-observer variability < 5% for kidney and liver dosimetry and supporting large-scale multicenter reproducibility [18, 34]. Together, these quantitative outcomes demonstrate that prospective, dosimetry-guided protocols enhance safety, reproducibility, and clinical throughput.

### Clinical Case Studies and Exemplars

Personalized dosimetry has begun to transform patient care in nuclear medicine by enabling risk-adapted, biologically informed, and outcome-driven therapies. In PRRT, the implementation of individualized kidney and bone marrow dosimetry allows dynamic risk stratification, minimization of organ toxicity, and tailored dose escalation in selected patients [30]. SIRT, utilizing voxel-based dosimetry, has enabled more precise tumor targeting and protection of normal hepatic parenchyma, particularly in patients with multifocal or complex hepatic lesions [47]. For alpha-particle therapies (e.g., ²²³Ra, ²²⁵Ac), micro-dosimetry and biological endpoint modeling are crucial to optimizing tumor control while preserving healthy tissue integrity [109, 110]. AI-driven automation is rapidly transitioning from research settings to clinical applications. Recent studies demonstrate that deep learning-based segmentation and absorbed dose mapping substantially reduce workflow time and variability, enabling real-time adaptive planning and improving reproducibility across operators and centers [102]. These advances not only enhance operational efficiency but also promote higher consistency and accuracy in patient-specific dose estimation, ultimately contributing to improved clinical outcomes [111].

### Clinical Trials and Practice Guidelines

The integration of patient-specific dosimetry and AI-augmented workflows is increasingly endorsed by major clinical trials and international guidelines, reflecting a paradigm shift toward personalized radiopharmaceutical therapy. Pivotal studies such as NETTER-1 [112] and DOSISPHERE-01 [113] have demonstrated that individualized dosimetry significantly improves tumor control while reducing toxicity, compared to conventional fixed-activity approaches, particularly in neuroendocrine tumors and hepatocellular carcinoma. In response, professional organizations including the EANM, Society of Nuclear Medicine and Molecular Imaging (SNMMI), and the International Atomic Energy Agency (IAEA) now recommend routine implementation of personalized dosimetry in both clinical trials and standard care. Concurrently, regulatory frameworks are evolving to support this transition, with a growing number of theranostic agents requiring dosimetric endpoints and individualized patient outcome data for approval and post-marketing surveillance. These developments underscore the critical role of quantitative dosimetry in enhancing both the therapeutic efficacy and safety of targeted radionuclide therapies.

### Quantitative Impact and cost-benefit Analysis

The clinical implementation of automated, AI-based dosimetry platforms offers measurable benefits across operational, clinical, and economic domains. Workflow studies have reported time savings of up to 80% compared to manual methods, along with significant improvements in reproducibility and reductions in operator-dependent error rates [114]. From a patient-centered perspective, dosimetry-guided therapy has been shown to achieve higher objective tumor response rates, lower frequency and severity of adverse events, and improved quality of life compared to fixed-activity dosing strategies [115]. From a health economics standpoint, although the adoption of advanced dosimetry platforms requires upfront investment in software, personnel training, and data infrastructure, these costs are often offset by reductions in toxicity-related hospitalizations, more efficient resource utilization, and the potential for improved long-term outcomes. Importantly, the democratization of dosimetry through user-friendly interfaces and AI-driven automation is expanding access to personalized dosimetry, even in smaller or resource-limited clinical settings.

### Future Directions and Patient Impact

The ongoing clinical translation of biology-informed and AI-driven dosimetry marks a paradigm shift in nuclear medicine, ushering in a new era of precision therapy in which individualized dose prescriptions are designed to maximize therapeutic efficacy while minimizing toxicity. As these strategies become integrated into routine clinical practice, future patients are expected to benefit from safer, more effective, and more equitable access to advanced radiopharmaceutical therapies.

## Toolkits and Dosimetry Software Platforms

### Toolkit for Clinical and Research Dosimetry Implementation

A growing number of commercial and academic software solutions are now available to support both clinical and research applications of dosimetry. These platforms accommodate a variety of imaging modalities, calculation methodologies, and workflow configurations, reflecting the diverse needs of modern nuclear medicine practices. The selection of an appropriate dosimetry platform should be guided by the specific clinical context, regulatory and compliance considerations, available institutional resources, and the desired level of automation or AI integration. Table 2 provides an overview of leading dosimetry software tools, highlighting their core features, supported dosimetric methodologies, and current regulatory status.

**Table 2:** Overview of leading dosimetry tools

Software Name	Developer/ Type	Methods Supported	Imaging Inputs	AI Integration	Regulatory Status	Notable Features	Website/Link
OLINDA/ EXM	Vanderbilt/ Commercial	MIRD, organ-level	SPECT, PET	No	FDA cleared	Widely validated, fast	https://www.hermesmedical.com/our-software/dosimetry/olinda/
PLANET Dose	DOSIsoft/ Commercial	Voxel-based, MC	SPECT, PET, CT	Some ML tools	CE marked	Voxel mapping, workflow	http://dosisoft.com/
STRATOS	Philips/Commercial	Voxel, MC	SPECT, PET, CT	Limited	CE marked	Auto-registration, multi-patient	http://philips.com/
MIRDcalc	Free/ Academic	MIRD, organ-level	SPECT, PET	No	Research	Web-based, user friendly	https://mirdsoft.org/mirdcalc
QDOSE	ABX/ Commercial	Voxel, organ, MC	SPECT, PET, CT	Some ML	CE marked	3D dosimetry, multi-tracer	https://qdose-plus.com/

*MC:* Monte Carlo; *ML*: Machine Learning; *MIRD*: Medical Internal Radiation Dose; *FDA*: U.S. Food and Drug Administration; *CE*: Conformité Européenne; *PET*: Positron Emission Tomography; *SPECT*: Single Photon Emission Computed Tomography; *CT*: Computed Tomography

### Practical Guide to Dosimetry Toolkits and Platform Selection

Several dedicated toolkits are now available for personalized dosimetry, each tailored to different clinical and computational needs. For ^177^Lu-based PRRT (DOTATATE or DOTATOC), organ-level assessment can be efficiently performed using OLINDA/EXM 2.0 [116], which remains the most widely validated reference software for standard kinetic analysis and regulatory reporting. When voxel-level quantification is required, platforms such as PLANET Dose (DOSIsoft), MIM SurePlan™ MRT, and the open-source VoxelDos or SlicerRT modules provide full 3D dose reconstruction and region-based statistical outputs for kidneys and tumors [96]. In PSMA-targeted radioligand therapy, voxelized systems such as STRATOS, Dosimetry Toolkit (GE Healthcare), or 3D Slicer extensions are preferred because they integrate semi-automated segmentation, multi-organ registration, and kernel convolution for voxelwise dose estimation. For ^90^Y-SIRT, SimIND, Planet Dose, and QDOSE™ allow dose reconstruction from ^90^Y PET or bremsstrahlung SPECT datasets, with liver-lung shunt correction and multicompartment dose partitioning [117].

In low-resource or regional centers, patient-specific dosimetry can be achieved using open-access software such as IDAC-Dose 2.1, VoxelDos, or 3D Slicer with SlicerRT, implemented on a standard workstation equipped with an Intel i7 or equivalent CPU, 16 GB RAM, and optional GPU acceleration [118]. When coupled with a well-calibrated SPECT/CT or PET/CT scanner and proper dose calibrator cross-validation, these platforms can achieve organ-level dose estimates within 10–15% of those obtained using commercial systems. This demonstrates that clinically meaningful, personalized dosimetry can be implemented even in settings with limited infrastructure, provided standardized RC and calibration procedures are maintained. The progressive availability of open data formats, harmonized phantom datasets, and low-cost computational platforms is expected to further expand the accessibility of quantitative dosimetry and reduce disparities in radiopharmaceutical therapy implementation worldwide.

## Literature Search on ¹⁷⁷Lu-PRRT Dosimetry (2020–2025)

### Search Strategy and Study Selection

**Fig. 3:**
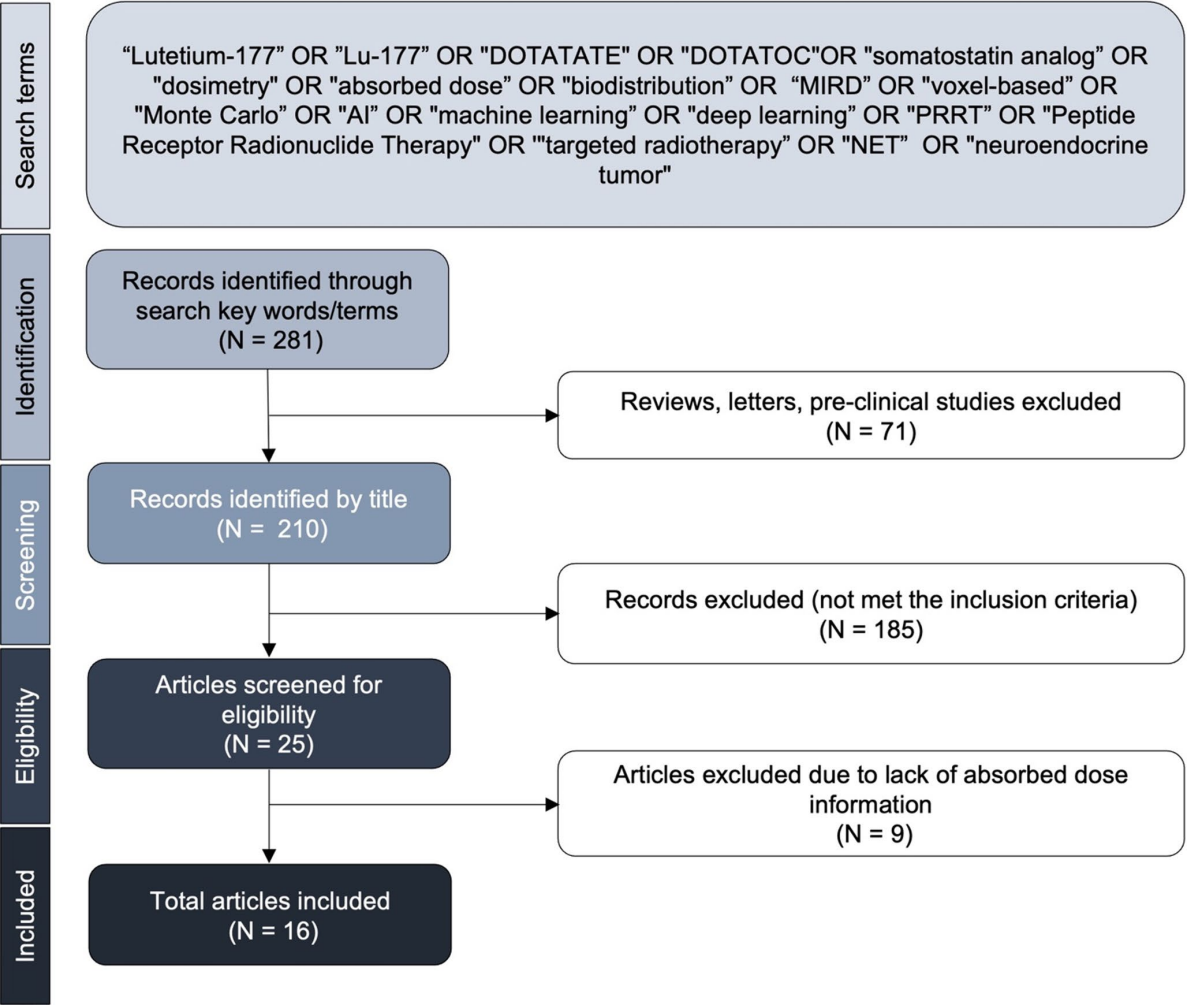
Schematic of the literature search methodology

A comprehensive literature search was conducted on PubMed for studies published between January 2020 and May 2025. The search terms were designed to comprehensively capture studies related to ¹⁷⁷Lu-PRRT dosimetry. A schematic overview of literature search methodology is presented in Fig. 3.

Human studies were included if they involved patients undergoing PRRT with ^177^Lu-labeled peptides such as DOTATATE, DOTATOC, or their variants. Eligible studies were required to provide quantitative absorbed dose estimates (expressed in Gy/GBq) for at least one organ or tumor site. All dosimetry methodologies were considered, including conventional MIRD or OLINDA-based approaches, as well as advanced voxel-based, MC, S-value kernel, or AI-assisted segmentation techniques. Studies were further required to specify the number of patients or lesions analyzed and to report the time points or methodology used for dose calculation.

Exclusion criteria comprised non-human studies (animal or in vitro), review articles, editorials, case reports, and methodological papers lacking patient-level dosimetry data. Studies that did not report absorbed dose values or provided only qualitative or visual uptake information without quantitative dosimetric assessment were also excluded.

### Data Extraction and Standardization

A total of sixteen eligible studies were included after full-text review for ^177^Lu-PRRT dosimetry. For each study, comprehensive information was extracted to ensure methodological transparency and reproducibility. The data included the year of publication, peptide used, injected activity (GBq), tumour type, and organs evaluated, along with scanner vendor and model, attenuation and scatter-correction details (CTAC, TEW), and reconstruction algorithm parameters such as OSEM iterations and subsets, post-filtering, and resolution-recovery (RR) settings. Information on the presence or absence of PVE correction or RC calibration and the method of VOI delineation-manual, semiautomatic, or AI-assisted-was also collected. For each study, absorbed dose values (Gy/GBq), measurement type (mean or median), number of subjects or lesions, standard deviation or interquartile range (SD/IQR), imaging time points, and any reported toxicity data were tabulated. Voxel- and SPECT-based dosimetry methodologies were documented in detail to enable accurate classification of MIRD, voxel-kernel, and MC-based approaches. Values annotated with asterisks were clarified in footnotes, and all abbreviations were defined accordingly. These expanded technical parameters are presented in Table 3.

**Table 3:** Summary of dosimetry studies on ¹⁷⁷Lu-PRRT in neuroendocrine tumors (NETs)

Year	Peptide	Injected Activity (GBq)	Tumor Type	Organ	Dosimetry Method	Scanner & other details	Dose (Gy/GBq)	Measure Type	*N*	SD/IQR	Time Point Imaging	Ref
2025	^177^Lu-DOTATATE	7.4 (not explicit)	NET	Kidney	Voxel (S-value kernel dosimetry via MIM SurePlan)	Siemens Symbia T2 and Veriton 200 (Spectrum Dynamics) CTAC: Yes Scatter: Yes (TEW) Reconstruction: OSEM 10 × 8 vs. 4 × 8 + RR PVE: Yes (phantom RC fit) VOI: AI TotalSegmentator + manual check	0.45 (conv), 0.47 (CZT); [0.25–0.79]	Median (min–max)	15	NA	24, 96, 168 h	[119]
				Spleen			0.48 (conv), 0.50 (CZT); [0.31–1.41]					
				Bone marrow			0.09 (conv), 0.13 (CZT); [0.04–1.96]					
				Liver			0.23 (conv), 0.21 (CZT); [0.05–0.72]					
				Tumor (largest)			1.8 (conv), 2.0 (CZT); [0.9–7.3]					
2025	^177^Lu-DOTATATE	3.687 ± 1.545 (1.469–7.368)	Pediatric neuroblastoma	Whole body	MIRD/Organ-level (HERMES Hybrid Dosimetry)	Siemens Symbia T16 SPECT/CT CTAC: Yes Scatter: Yes (dual-window) Reconstruction: OSEM 8 × 6 + Butterworth filter (0.5, 5) PVE: No (not applied) VOI: Planar + SPECT-based semi-automatic ROIs; marrow from vertebral region	0.159	Mean ± SD	23	0.076	0,1,2,4,6,24,48,96 h	[120]
				Red marrow			0.611			0.416		
2025	^177^Lu-DOTATATE	7.21	NET	Kidney	MIRD/organ-level (primary) Task 5 TIA map generated by voxelwise fitting in MIM with AIC model selection; participants then applied VOIs and converted TIAs to AD with MIRDcalc 1.1	NA	0.291	Single case	1	NA	4 TP (3.7–193.3 h)	[121]
				Kidney			0.57					
				Lesion			5.04					
				Lesion			1.22					
2025	^177^Lu-DOTATATE	26.2 (6.6) cumulative (mean)	Midgut NET	Kidney	MIRD planar dosimetry Tumor doses derived from OLINDA sphere model, interpolated by lesion mass.	No scanner model specified Planar imaging (geometric mean) for all organ and tumor dosimetry, with SPECT/CT only for visual confirmation. CTAC and scatter correction followed standard MIRD procedures (per Pamphlet 16). No PVE correction, since only planar quantification was used.	0.65 (0.30) mean	Mean (SD) & Median (range)	20	0.30 (kidney)	Planar 4–6 TPs, SPECT/CT at 24/48 h	[112]
							0.68 (0.16–1.17) median (range)					
				Liver			0.30 (0.23) mean		18	0.23 (liver)		
							0.33 (0.05–2.06) median (range)					
				Red marrow			0.04 (0.03) mean		20	0.03		
							0.03 (0.01–0.14) median (range)					
				Spleen			0.85 (0.80) mean		20	0.8		
							0.62 (0.18–3.61) median (range)					
				Tumor (lesion)			7.6 (10.6) mean		65*	10.6		
							4.5 (0.2–74.9) median (range)					
2025	^177^Lu-DOTATATE	7.4 (×4 cycles)	NET	Kidney	MIRD (OLINDA organ and sphere models)	Siemens Symbia Intevo Bold SPECT/CT CTAC: Yes Scatter: Yes (dual energy windows 10%) Reconstruction: OSEM 24 × 4 + Gaussian 8.4 mm filter PVE correction: Yes (phantom recovery coefficients applied) VOI definition: Manual (ImageJ threshold from NEMA phantom)	1st cycle: 3 Gy 4th cycle: 3 Gy	Median	17	1st cycle: 2–3 Gy 4th cycle: 3–4	1, 3, 7 days (cycle 1,4)	[122]
				Lesion			1st cycle: 21 Gy 4th cycle: 14 Gy	Median	64**	1st cycle: 14–29 Gy 4th cycle: 8–19 Gy	1, 3, 7 days	
2024	^177^Lu-DOTATATE	4 × 7.4 (29.6)	GEP NET (G1/G2)	Tumor (GTD)	MIRD/OLINDA organ-level, prognostic GTD model	Siemens Symbia T16 SPECT/CT CTAC: Yes Scatter: Yes (dual window) Reconstruction: OSEM 24 × 4 + Gaussian 8 mm filter PVE corr: No VOI: Manual on fused SPECT/CT, CT mass weighting per lesion	GTD₁ cut-off 10.6 Gy, HR = 8.6 (95% CI 2–37), *p* = 0.004. GTDₜₒₜ cut-off 43 Gy, *p* = 0.035. 1.45 (cutoff)	Prognostic threshold	42	NA	1, 7 days post-inj (cycle 1 & 4)	[123]
2024	^177^Lu-SSTR-RT (agonist)	7.4/cycle (typical)	Meningioma	Tumor	Predominantly MIRD (organ or lesion-level), a few voxel-based	Mixed (Siemens, GE, Philips - no single model) CTAC: Yes (reported in most). Scatter: Yes (dual/TEW). Reconstruction: OSEM 4–10 × 6–8 + Gaussian filter (varied). PVE correction: Rare; only one study applied RC. VOI definition: Manual SPECT/CT or MRI co-registration	0.1–1.5 (majority: 0.1–1.5)	Mean/range	46 pts/108 cycles	0.07–13.3	2–5 TPs (most); some 1 TP	[124]
							1.0–5.5		6 pts	1.0–5.5	3 TPs (recommended)	
2024	^177^Lu-DOTATATE	22–67 (median 45, G1); 15–45 (median 30, G2)	NET (G1, G2)	Tumor	Hybrid planar + SPECT/CT with voxel-based absorbed-dose-rate calculation	PVE correction: Yes VOI rules: Semiautomatic segmentation; manual CT delineation; exclude < 4 cm³; must be quantifiable on baseline CECT & hybrid images	4.0 (G1)3.6 (G2)4.5 (90% TCP, G2)	Median	32/69	NA	SPECT/CT + planar, multi-TP/cycle	[125]
2024	^177^Lu-DOTATATE	4 × 7.4 (29.6)	GEP-NET (G2)	Kidney	Hybrid voxel + organ (MIRD + local ED method)	GE Discovery NM/CT 670 SPECT/CT CTAC: Yes Scatter: Yes (10% window 177 keV) Reconstruction: OSEM quantitative (attenuation + scatter + RR + density corr.) PVE: Yes (phantom RC) VOI: Manual CT-based; ≤ 5 lesions per site; exclude < 2 cm³	10.77 / 29.6 = 0.36	Median	35	(4.99–21.12) Gy	4 TP (4,24,72,192 h, cycles 1&2), 1 TP (24 h, 3&4)	[3]
				Spleen			14.28 / 29.6 = 0.48			(6.87–23.87) Gy		
				Bone Marrow			1.07 / 29.6 = 0.036			(0.63–3.74) Gy		
				Tumor			94.43 / 29.6 = 3.19		35 / 146 lesions	(8.73–287.89) Gy		
2024	^177^Lu-DOTATATE	4 × 7.4 (mean 7.3/cycle)	NET (histologically confirmed)	Red Marrow	Monte Carlo microscale & macroscale DPM dosimetry	Siemens Intevo Bold SPECT/CT (LEHR collimator) CTAC: Yes (120 kVp 80 mAs reference; 15 mAs for others). Scatter: Yes (TEW). Reconstruction: OSCG 48 × 1 with resolution recovery, no filtering. PVC: No explicit RC-based PVC applied (quantitative validated pipeline). VOI: AI-based vertebral spongiosa segmentation via nnU-Net + manual QA	0.049 (0.019–0.11)	Median (range)	20	0.049 ± 0.019	0, 1, 4–5, 5–8 days post-inj, 4 cycles	[126]
2023	^177^Lu-DOTATATE	7.4 (standard, cycle 1)	GEP-NET	Kidney	Monte Carlo (voxel-level)	Siemens Intevo Bold SPECT/CT; Siemens Biograph mCT PET/CT CTAC: Yes. Scatter: Yes (TEW dual-window). Reconstruction: Quantitative OSEM 8 × 8 + RR. PVC: No (validated quantitative calibration). VOI: DL segmentation + manual QC	0.5 (0.2–1.0)	-	25/50 kidneys	-	4, 24, 96, 168 h	[127]
2022	^177^Lu-DOTATATE	7.6 (6.1–8.0) per cycle	NET (various; 29 SI, 5 PNET, 5 other; G1–G3)	Kidney	Hybrid MIRD/MCNP6 ACDF Monte Carlo	GE Discovery NM/CT 670 SPECT/CT CTAC: Yes (low-dose 120 kV CT). Scatter: Yes (TEW). Reconstruction: OSEM 6 × 10 + RR + Butterworth 0.4/5. PVC: No; quantitative via multi-VOI averaging. VOI: Manual small 0.6 mL ROIs; 42% threshold; ≥ 22 mm tumors; tracked across cycles.	0.42 (0.32–0.54) (median, all cycles)	Median	39 pts / 147 cycles / 291 kidneys	0.28	1 d & 7 d SPECT/CT	[128]
			NET (see above)	Tumor			1.75 (0.73–2.90) (median, all cycles)		284 tumors	1.32 (tumor)		
			Small Intestine NET (by grade)	Tumor (G1)			2.15 (1.42–2.83)		9 pts (G1), multi-cycle	1.42–2.83		
			Small Intestine NET (by grade)	Tumor (G2)			1.09 (0.70–1.83)		16 pts (G2), multi-cycle	0.70–1.83		
2021	^177^Lu-DOTATATE	7–8 per cycle (typical)	NET	Kidney	MIRD + voxel hybrid (MIM SurePlan)	Siemens Symbia T16 SPECT/CT CTAC: Yes Scatter: Yes (TEW) Reconstruction: OSEM 12 × 8 + RR + Butterworth 0.45/5 PVE correction: Yes (RC-based from NEMA phantom) VOI definition: Manual SPECT/CT; lesions ≥ 1 mL	0.47 (0.20–1.19)	Median, Mean	56/56 fractions	0.13	1, 4, 7 d	[129]
	^177^Lu-DOTATOC						0.37 (0.19–0.78)	Median, Mean	59/59 fractions	0.13		
2022	^177^Lu-DOTATOC	2.33 ± 0.52 (1.59–3.49)	NET	Tumor	Hermes HIRD + OLINDA/EXM 2.0 MIRD-based hybrid	GE Discovery NM 670 SPECT/CT CTAC: Yes (low-dose CT). Scatter: Yes (TEW). Reconstruction: OSEM 4 × 8 + Hanning 0.85 cycles/cm. PVC: None (explicit); quantitative calibration using standard source. VOI: Manual ROIs on planar + SPECT/CT, background subtraction applied.	1.29 ± 0.86 (0.27–3.23)	Mean (range)	8	0.86	0.5, 24, 48, 72 h planar; 24 h	[130]
				Spleen			0.46 ± 0.41 (0.06–1.18)			0.41		
				Kidney			0.38 ± 0.11 (0.28–0.65)			0.11		
				Red Marrow			0.03 ± 0.01 (0.01–0.05)			0.01		
2021	^177^Lu-DOTA-LM3	6.1 ± 0.8 (2.8–7.4)	Metastatic NENs	Kidney	MIRD (OLINDA v1.1 planar + SPECT/CT hybrid)	Mediso Spirit DH-V dual-head SPECT/CT CTAC: Yes. Scatter: Yes (15% 208 keV window + adjacent scatter windows). Reconstruction: Planar geometric mean + quantitative SPECT/CT (24 h). PVC: No. VOI: Manual Bad Berka protocol (Mono/Bi-exp fits)	2.3 ± 0.9 (0.5–3.6)	Mean	11	0.9 (kidney)	0.5,2.5,19.5,44,68.5,118 h	[131]
				Spleen			3.4 ± 1.6 (1.2–5.4)	Mean		1.6 (spleen)		
				Liver			0.39 ± 0.05 (0.35–0.44)	Mean		0.05 (liver)		
				Tumor (liver mets)			15–81 (mean: 51, up to 93)	Range / Mean		(15–81, mean 51)		
				Tumor (bone mets)			1–57 (mean not stated)	Range		(1–57)		
2020	^177^Lu-DOTATOC	6.53 ± 0.45	GEP-NETs (G1–G3)	Kidney	Monte Carlo voxel-based (TTD computed per Eq. ΣD_i_·V_i_ / ΣV_i_)	Siemens Symbia T2 SPECT/CT (MEGP collimator) CTAC: Yes (low-dose CT for AC). Scatter: Yes (TEW). Reconstruction: OSEM 16 × 8 + PSF, no smoothing. PVE corr.: No explicit RC correction (quantitative validated pipeline). VOI definition: Manual on fused SPECT/CT; outlier rejection via alpha-stable distribution.	0.52 ± 0.20 (0.30–0.97)	Mean	14	0.20	4 h, 24 h, 48 h, 72 h (4 TP)	[107]
				Spleen			0.67 ± 0.41 (0.20–1.62)			0.41		
				Tumor			1.46 ± 1.26 (0.20–5.60)			1.26		

*Number of lesions, not patients; n = 17 patients for tumor dosimetry, **64 total lesions in 17 patientsAbbreviations: AI = Artificial Intelligence; CZT = Cadmium Zinc Telluride; CT = Computed Tomography; CTAC = CT-based Attenuation Correction; ED = Energy-Dependent; FOV = Field of View; GEP = GastroEnteroPancreatic; GTD = Gross Tumor Dose; Gy/GBq = Absorbed dose per injected activity (Gray per gigabecquerel); IQR = Interquartile Range; MC = Monte Carlo; MIRD = Medical Internal Radiation Dose; MTP = Multi-Time-Point imaging protocol; NA = Not Available; NET = Neuroendocrine Tumor; OSEM = Ordered Subset Expectation Maximization; OLINDA = Organ Level INternal Dose Assessment; PET = Positron Emission Tomography; PFS = Progression-Free Survival; PVC = Partial Volume Correction; PVE = Partial Volume Effect; RC = Recovery Coefficient; RR = Resolution Recovery; SD = Standard Deviation; SPECT = Single Photon Emission Computed Tomography; STP = Single-Time-Point imaging protocol; TCP = Tumor Control Probability; TEW = Triple Energy Window; TP = Time Point; VOI = Volume of Interest; n = Number of patients or lesions (as indicated in context); Cycle = One administration of the radiopharmaceutical treatment; Teff = Effective half-life

### Analysis

The primary endpoint was the comparison of absorbed dose estimates across different dosimetry methods, imaging time-point schemes, and organs, including the kidney, liver, spleen, red marrow, and tumors.

### Results

In this literature search of ¹⁷⁷Lu-PRRT studies published between 2020 and 2025 (Table 3), 16 studies reporting patient-level dosimetry for kidneys, liver, red marrow, spleen, and tumors were evaluated. A clear methodological shift was observed from traditional MIRD-based organ-level dosimetry (e.g., OLINDA/EXM) to voxel-based approaches incorporating MC simulations and voxel S-value kernels. These advanced methods enhance spatial accuracy and enable personalized dose mapping, especially for tumors and red marrow. Recent studies have also introduced AI-assisted segmentation and Cadmium-zinc telluride (CZT)-based SPECT imaging, improving automated workflows and organ delineation, though validation remains limited.

Imaging protocols varied widely, from single time-point to MTP schemes, with MTP SPECT/CT remaining the gold standard for capturing pharmacokinetics. Tumor absorbed doses showed the highest variability (1.2–7.6 Gy/GBq, with some lesions exceeding 90 Gy/GBq), reflecting biological heterogeneity and methodological sensitivity. Kidney doses were consistently reported between 0.3 and 0.6 Gy/GBq, with spleen and liver showing intermediate uptake. Red marrow dosimetry, typically estimated via blood-based methods and AI-guided MC simulations, generally yielded doses below 0.06 Gy/GBq. Despite methodological advances, challenges persist, including lack of protocol harmonization, limited use of individualized biokinetics, and insufficient correlation of dosimetry with therapeutic response or toxicity. These findings emphasize the urgent need for standardized, AI-integrated voxel-based dosimetry frameworks to enable routine personalized treatment planning in nuclear medicine.

#### Dosimetry Methodology and Comparative Summary

For each study, scanner-specific and reconstruction-related parameters-including CTAC, scatter-correction schemes, OSEM iteration-subset configurations, partial-volume or RC corrections, and VOI delineation rules-were extracted to ensure reproducibility. Studies were categorized into MIRD/OLINDA organ-level (planar or hybrid planar + SPECT/CT), voxel-based kernel or S-value methods, and MC or hybrid MC-based voxel dosimetry frameworks. Hybrid workflows combining planar and voxel-level calculations or applying local energy-deposition corrections were classified separately. Imaging protocols were additionally labelled as STP or MTP depending on the number of quantitative acquisitions performed per treatment cycle. Quantitative results were summarized as per-study medians, IQRs, and ranges for each organ and tumour site. When at least three studies reported comparable data, pooled medians [IQR] were calculated; otherwise, representative ranges were provided. These grouped comparisons by dosimetry method (MIRD, Voxel, MC) and temporal sampling strategy (STP vs. MTP) are presented in Table 4. Overall, methodological heterogeneity was evident, yet a consistent shift toward voxel and MC dosimetry has emerged. As shown in Table 4, kidney doses were similar across approaches-MIRD: 0.47–0.68 Gy/GBq, voxel-based: 0.47 [0.32–0.54] Gy/GBq, and MC-based: 0.50 [0.42–0.52] Gy/GBq-while spleen and liver absorbed doses were intermediate and red marrow values generally remained below 0.1 Gy/GBq. Tumour or lesion doses showed the largest variability (median = 1–7 Gy/GBq, with extremes > 90 Gy/GBq), influenced by lesion size, biological heterogeneity, and segmentation strategy. Application of partial-volume correction or RC calibration increased lesion-dose estimates by approximately 10–15%, whereas omission led to underestimation in small volumes. AI-based segmentation tools such as TotalSegmentator and nnU-Net improved VOI reproducibility and reduced observer bias. STP dosimetry, validated against multi-time-point models within MC frameworks, showed deviations within ± 3% of integrated values, supporting its potential for clinical streamlining. Collectively, voxel and MC methodologies provide superior accuracy and consistency compared to planar MIRD-based methods, particularly for heterogeneous tumours and bone marrow. Incorporating standardized reconstruction parameters, multi-time-point sampling, and AI-assisted segmentation will be critical for the next generation of personalized ^177^Lu-PRRT dosimetry.

**Table 4:** Summary of reported organ- and lesion-level absorbed doses (Gy/GBq) from ^177^Lu-labeled peptide therapies, grouped by dosimetry method and sampling type

Dosimetry Method	Sampling Type	Kidney	Spleen	Liver	Red Marrow	Tumor / Lesion
MIRD / OLINDA (planar or hybrid)	MTP	0.47–0.68 (study medians)	0.46–0.85	0.30	0.03	1.29–7.6
Voxel-based (S-value / hybrid)	MTP	0.47 [0.32–0.54]	0.50 [0.30–0.67]	0.23 [0.05–0.72]	0.036–0.13	1.7 [0.7–2.9]
Monte Carlo (MC or hybrid MC)	MTP	0.50 [0.42–0.52]	0.67 [0.41–1.62]	0.39 [0.35–0.44]	0.049 [0.019–0.11]	1.46 [0.70–2.90]
MIRD / MC hybrid (simplified)	STP	0.42 [0.32–0.54]	–	–	–	1.75 [0.73–2.90]
Other (SSTR-RT in meningioma)	MTP (mixed)	–	–	–	–	0.1–1.5

Values represent study-level medians [IQR] or ranges when fewer than three studies contributed.All data are derived from publications between 2020 and 2025 and standardized to Gy/GBq for comparison. Abbreviations: MIRD = Medical Internal Radiation Dose; OLINDA = Organ Level INternal Dose Assessment; MC = Monte Carlo; SSTR-RT = Somatostatin Receptor–Targeted Radionuclide Therapy; STP = Single-Time-Point; MTP = Multi-Time-Point; RC = Recovery Coefficient; PVE = Partial-Volume Effect; VOI = Volume of Interest; Gy/GBq = Absorbed dose per injected activity

## Current Challenges and Future Roadmap

### Standardization and Reproducibility

A major barrier to the widespread clinical adoption of personalized dosimetry remains the lack of standardized procedures and protocols across institutions. Variability in imaging techniques, segmentation methods, dose calculation approaches, and reporting formats compromises reproducibility and limits result comparability [34]. Although international harmonization efforts led by the EANM, SNMMI, and IAEA are ongoing, stronger adherence and continuous updates are needed to keep pace with emerging technologies.

### Data Integration and Multimodal Complexity

Dosimetry is increasingly evolving toward the integration of physical measurements, biological markers, and AI-derived data, which substantially amplifies the complexity of data management and interpretation. However, the absence of interoperable data standards and insufficient hospital IT infrastructure present significant barriers to the seamless transfer of information from imaging acquisition through therapy planning to clinical follow-up. To overcome these challenges, the development of scalable and flexible data architectures is imperative. Furthermore, adherence to FAIR (Findable, Accessible, Interoperable, Reusable) data principles and the establishment of secure, privacy-compliant data-sharing frameworks constitute urgent priorities to enable efficient, reproducible, and collaborative dosimetric workflows.

### Biological Dosimetry and Biomarker Validation

Although biological endpoints such as γ-H2AX, circulating DNA, and transcriptomic signatures hold promise for enhancing dose-response modeling, robust and clinically validated biomarkers remain limited [132]. To establish their clinical utility, multi-center collaborations, standardized biobanking protocols, and well-designed prospective trials are essential. Such coordinated efforts will be critical to validate these biomarkers and ensure their reliability for routine clinical implementation.

### AI/ML Challenges: Data scarcity, validation, and Trust

The rapid proliferation of AI and machine learning models in dosimetry is constrained by the limited availability of large, well-annotated, and diverse datasets necessary for robust model training and external validation [133–135]. The majority of existing models have been assessed using single-center, retrospective datasets, raising significant concerns regarding their generalizability across broader clinical settings. Moreover, the inherent opacity of deep learning algorithms - the so-called “black box” phenomenon [136]-poses challenges for clinician acceptance and regulatory approval. To address these issues, progress in explainable AI techniques, standardized reporting guidelines, and the establishment of multicenter validation frameworks will be critical.

## Conclusion

The next decade will see the convergence of standardized, multimodal data integration, robust biomarker-driven biology, and trustworthy, explainable AI. Priorities include: harmonizing standards and guidelines internationally, establishing large, shared datasets for model training and validation, developing validated biological endpoints for patient-specific response assessment, promoting transparency, explainability, and clinician engagement in AI, embedding personalized dosimetry into regulatory and reimbursement frameworks. Collaborative, interdisciplinary partnerships across clinical, academic, industry, and regulatory domains will be essential to achieve these goals and realize the promise of next-generation personalized radiopharmaceutical therapy. 

## Data Availability

Not applicable.

## Declarations

### Declarations of generative AI in scientific writing

Authors did not use generative AI in writing except for checking grammar, spelling, references, etc.
